# Editorial: Ferroptosis as a novel therapeutic target for inflammation-related diseases

**DOI:** 10.3389/fphar.2023.1152326

**Published:** 2023-02-16

**Authors:** Yongyi Liang, Zhenyi Su, Xiaoyuan Mao, Shibiao Wan, Lianxiang Luo

**Affiliations:** ^1^ The First Clinical College, Guangdong Medical University, Zhanjiang, China; ^2^ Herbert Irving Comprehensive Cancer Center, Vagelos College of Physicians and Surgeons, Columbia University Irving Medical Center, New York, NY, United States; ^3^ Department of Clinical Pharmacology, Xiangya Hospital, Central South University, Changsha, China; ^4^ Department of Genetics, Cell Biology and Anatomy, University of Nebraska Medical Center, Omaha, NE, United States; ^5^ The Marine Biomedical Research Institute, Guangdong Medical University, Zhanjiang, China; ^6^ The Marine Biomedical Research Institute of Guangdong Zhanjiang, Zhanjiang, China

**Keywords:** ferroptosis, inflammation-related diseases, lipid peroxidation, inflammatory mediators, immunomodulatory

Ferroptosis was first proposed in 2012 as a relatively new mode of cell death (Dixon et al., 2012). It is driven by iron-dependent lipid peroxidation, which is a programmed cell death distinct from apoptosis, various forms of necrosis, and autophagy. At the ultrastructural level, ferroptotic cells usually exhibit mitochondrial abnormalities (Tang et al., 2021). There are shared features in ferroptosis and inflammatory diseases, including Depletion of glutathione peroxidase four and glutathione, elevated lipid peroxidation products, as well as disrupted iron metabolism (Mao et al., 2020). As the process of a systemic response, inflammation can be protective or pathological. Inflammatory biology is associated with almost all human diseases (Medzhitov, 2021).

Ferroptosis is closely related to inflammation. Traces of inflammation can be easily captured in pathologies involving ferroptosis (Stockwell, 2022). Ferroptosis is accompanied by the release of proinflammatory molecules, such as interleukin (IL)-1β and IL-18 (Sun et al., 2020). Meanwhile, ferroptosis exacerbates the inflammatory response to varying degrees through mediators such as ferroptosis regulators like glutathione peroxidase 4, reactive oxygen species, lipoxygenases, and inflammatory mediators produced during ferroptosis (Deng et al., 2022). Several anti-inflammatory drugs have been shown to inhibit ferroptosis in certain cellular models (Wang et al., 2023). However, studies exploring the role of ferroptosis in inflammation are still scarce, though the research field of ferroptosis has been enjoying exponential growth over the past few years (Jiang et al., 2021), especially in cancer treatment (Chen et al., 2021). Nanomaterials targeting ferroptosis-based cancer therapy have shown considerable promise (Luo et al., 2021). Our Research Topic includes four reviews and three original articles studying ferroptosis and inflammatory diseases, as well as related drugs that contribute to the regulation of ferroptosis for the treatment of inflammatory diseases.

Many inflammatory diseases are associated with ferroptosis, such as acute renal failure, Acute lung injury, neurodegenerative diseases, chronic autoimmune diseases of the gastrointestinal, *etc.* (Deng et al., 2022). A review by Zhang et al. reported that inflammatory-associated intestinal diseases are strongly associated with ferroptosis, indicating that ferroptosis may act as potential therapeutic targets for inflammatory-associated intestinal diseases. In addition, some inflammatory diseases are infectious, such as COVID-19. Iron overload is a contributing factor to COVID-19. If left untreated, ferroptosis can promote a range of responses that enhance inflammation, leading to multi-organ failure, lung injury, and reduced lung capacity (Habib et al., 2021). A review by Xiao et al. summarized the mechanisms of ferroptosis, elucidated the role of ferroptosis in the onset and progression of different infectious diseases. At the same time, it suggested that ferroptosis may be a new therapeutic target for the development of more effective adjuvant therapies for infectious diseases. Based the relationship between ferroptosis and inflammation, researchers have found that anti-inflammatory drugs can inhibit ferroptosis. It has been demonstrated that the anti-inflammatory drug quercetin may inhibit ferroptosis, *via* the PI3K/AKT/mTOR pathway (Lan et al., 2022). L-cit, which has anti-inflammatory properties, may target ferritin phagocytosis and mediate ferroptosis (Ba et al., 2022). A review by Li et al. described the active glycoside component of the medicinal plant *Anoectochilus roxburghii*, kinsenoside (KD), which also has potent anti-inflammatory and antioxidant properties. It summarized the multiple actions of KD and suggested that it may regulate ferroptosis. Another review by Zeng et al. investigated the effects of various anesthetics on molecular mechanisms and signaling pathways related to ferroptosis. The authors hypothesized that the mechanism of action of anesthetics varies across experiments and different cellular systems because of the paradoxical nature of anesthetics to inhibit ferroptosis in inflammatory disease models but promote ferroptosis in tumor cells. It may exhibit anti-inflammatory and anti-ferroptosis effects in inflammatory diseases such as IRI.

In the meantime, the three original articles in our Research Topic also focused on the study of iron death and inflammatory diseases and related drugs. Xu et al. predicted a signature for acute coronary syndrome (ACS) based on the expression levels of genes related to iron metabolism and identified novel serum iron gene markers in the early stages of ACS. Five genes, namely, PADI4, HLA-DQA1, LCN2, CD7 and VNN1, were selected by using a feature-selection method called Elastic Net and included in the final Immune Gene Signature model. Pan et al. used integrated bioinformatics to analyze ferroptosis, necroptosis and scorch-related genes in periodontitis-affected periodontitis tissues to obtain 21 differential genes, together with their associated cellular and immune pathways. Among them, SLC2A3 was associated with ferroptosis. The upregulation of SLC2A3 was positively correlated with periodontal neutrophil infiltration. Herbal medicine may have a significant role in the regulation of ferroptosis (Gao et al., 2022). HJ11 is a novel Chinese medicine derived from the appropriate addition and reduction of Si Miao Yong An Tang. Zhang et al. presented a model of myocardial ischemia-reperfusion (I/R) injury in rats and demonstrated that HJ11 decoction inhibits the development of myocardial I/R injury by regulating ACSL4-mediated ferroptosis. Thus, HJ11 soup may be an effective drug for the treatment of myocardial I/R injury.

In conclusion, ferroptosis is highly correlated with inflammation, for which one potential way to treat inflammatory diseases is based on regulating ferroptosis. The discovery and study of various drugs that modulate ferroptosis have brought new hope to the clinical treatment of inflammatory diseases. The studies in this Research Topic provide evidence for the important role of ferroptosis in the treatment of inflammation, elaborate the specific mechanisms of ferroptosis action in some diseases. It also shows that many ferroptosis-related genes and molecules can serve as key targets for disease treatment. Meanwhile, drug discovery and research have provided new directions for the regulation of ferroptosis. However, questions such as how to maximize the use of ferroptosis to minimize the harm to patients, how to grasp the time point of drug intervention and the exact dosage, and the different regulatory effects of drugs on ferroptosis in tumor cells and inflammatory cells are still waiting for researchers to explore.
